# Individual- and community-level factors associated with the presence of adequate iodized salt in households in Bangladesh: a multilevel modelling approach

**DOI:** 10.1093/inthealth/ihae016

**Published:** 2024-02-12

**Authors:** Iqramul Haq, Md Ismail Hossain, Md Rukonozzaman Rukon, Md Jakaria Habib, Tanha Akther Tithy, Md Amit Hasan, Salma Akter, Md Rayhan Ali Rejvi, M Sheikh Giash Uddin, Md Mizanur Rahman Sarker, Fasil Wagnew, Ashis Talukder

**Affiliations:** Department of Agricultural Statistics, Sher-e-Bangla Agricultural University, Dhaka-1207, Bangladesh; Department of Mathematics and Natural Sciences, BRAC University, Dhaka-1212, Bangladesh; Department of Statistics, Jagannath University, Dhaka-1100, Bangladesh; Department of Statistics, Jagannath University, Dhaka-1100, Bangladesh; Department of Statistics, Jagannath University, Dhaka-1100, Bangladesh; Department of Statistics, Jagannath University, Dhaka-1100, Bangladesh; Department of Statistics, Jagannath University, Dhaka-1100, Bangladesh; Department of Agricultural Economics, Sher-e-Bangla Agricultural University, Dhaka-1207, Bangladesh; Department of Statistics, Jagannath University, Dhaka-1100, Bangladesh; Department of Agricultural Statistics, Sher-e-Bangla Agricultural University, Dhaka-1207, Bangladesh; College of Health Sciences, Debre Markos University, Debre Markos, Ethiopia; National Centre for Epidemiology and Population Health, Australian National University, Canberra, ACT, Australia; Statistics Discipline, Khulna University, Khulna-9208, Bangladesh; National Centre for Epidemiology and Population Health, Australian National University, Canberra, ACT, Australia

**Keywords:** adequacy, hypothalamus, iodine, micronutrient, salt, T3, thyroid

## Abstract

**Background:**

The aim of this study is to estimate the factors at both the individual and community levels related to the adequacy of iodized salt in households in Bangladesh.

**Methods:**

For this study we utilized the 2019 Multiple Indicator Cluster Survey data. A total of 61 242 households were chosen as samples from Bangladesh. In our study of socio-economic disparities, we applied a concentration indexing method. To identify the factors associated with the adequacy of iodine in salt at both the individual and community levels, we employed multilevel logistic regression. Aside from the multilevel regression used in the study, we also applied spatial analysis.

**Results:**

The results indicated that the prevalence of iodine adequacy in household salt was found to be 57.8% (95% confidence interval 57.4 to 58.2). Rural areas have a higher concentration of iodine than urban areas. According to the multilevel model, younger women (adjusted odds ratio [aOR] 0.70), Muslim women (aOR 0.89), illiterate women (aOR 0.80) and those from poor households (aOR 0.33) were found to be less likely to consume iodine in concentrated salt compared with their counterparts. Disabled women and those with low media exposure have a lower likelihood of iodine adequacy in salt compared to their reference group. Furthermore, households in urban areas exhibited higher odds of having iodine adequacy in salt compared with households in rural areas. Barisal, Chattogram, Dhaka, Khulna, Mymensingh, Rajshahi and Rangpur Divisions have lower iodine adequacy in salt compared with Sylhet Division.

**Conclusions:**

The findings reveal that religion, physical disability and exposure to media exert an equal influence on the presence of iodized salt intake. Moreover, women's age, wealth status, education level and the educational background of the household head positively contribute to the adequacy of iodine in household salt. In light of these results, policymakers are advised to prioritize efforts aimed at enhancing iodine concentration, with a particular focus on mass media advertising, especially in rural areas (excluding Sylhet Division).

## Introduction

Iodine, an essential micronutrient, plays a critical role in the synthesis of thyroid hormones, including triiodothyronine (T3) and thyroxine (T4), which are vital for human growth, development and metabolic regulation.^1,2^ The World Health Organization (WHO) and United Nations Children's Fund (UNICEF) emphasize the importance of including iodine-rich foods in our diets to support optimal neurological function.^3^ Despite these efforts, iodine deficiency remains a global concern, resulting in iodine deficiency disorders (IDDs) that affect approximately 1.9 million people worldwide.^4^ IDDs can lead to conditions such as endemic goitre, brain damage, growth impairment and various health complications. To combat this issue, the WHO has launched universal salt iodization (USI) programs worldwide, making iodized salt consumption a mandatory strategy.^5^

Despite substantial global efforts to combat IDDs from 2003 to 2011, disparities persist, especially in low- and middle-income countries.^6^ For instance, Ethiopia continues to grapple with iodine deficiency as a major public health challenge,^7^ as recent studies there reveal concerning statistics: >60% of households lack adequately iodized salt, with even higher non-exposure rates among low-income families.^8^ Similarly, in Bangladesh, where government mandates promote iodized salt consumption, iodine deficiency rates remain persistently high.^9–11^ According to the 2019–2020 national micronutrient survey conducted by the International Centre for Diarrhoeal Disease Research, Bangladesh, iodine deficiency rates were reported at 19.7% in children and 29.6% in women.^12^ The 2015 Bangladesh National Salt Iodization Survey (NSIS) indicated that only 50.5% of households had access to adequately iodized salt, with 65.0% having any iodized salt at all.^11^ Unfortunately, progress in addressing these issues has been limited, with concerning declines noted in certain areas.

In 2014–2015, a study conducted among schoolchildren in Dhaka city unveiled alarming rates of iodine deficiency, affecting a staggering 83.2%.^13^ In addition, >2.2 billion individuals reside in iodine-deficient environments across 130 countries,^15^ with pregnant women facing a 50% increased daily iodine requirement, making them particularly susceptible to severe iodine deficiency.^16^ Prior investigations in rural Bangladesh employed various statistical models, such as binary logistic regression^14^ and Bayesian mixed effects logistic models,^11^ to delve into iodine insufficiency and its correlation with household iodized salt. These studies also sought to pinpoint contributing factors, including low dietary iodine intake, pregnancy, gender, tobacco use, alcohol consumption, age and radiation exposure. Despite these collective efforts, there remains a pressing need to investigate localized prevalence and assess inequality in iodized salt adequacy at the grassroots level in Bangladesh.

To bridge existing knowledge gaps, our study explores the individual- and community-level factors that influence the presence of adequate iodized salt in Bangladeshi households. Methodologically, we break from tradition by adopting a multilevel modelling approach, which proves to be a substantial improvement over single-level binary logistic regression models often used in binary response variable studies. This approach is particularly relevant when working with datasets characterized by hierarchical structures, where the conventional assumptions of observation independence and uncorrelated residual errors may not consistently hold. Drawing data from the Bangladesh Multiple Indicator Cluster Survey (MICS) dataset, which inherently embodies a hierarchical structure, our primary objective was to explore the determinants associated with the adequacy of iodized salt at the household level. Furthermore, we examine socio-economic disparities in urban and rural contexts within Bangladesh. Our research not only contributes significantly to the broader understanding of public health and nutrition in Bangladesh, but also holds critical importance in combating IDDs and enhancing the overall health of the population. Additionally, our findings can help inform governmental initiatives and align national objectives with international targets, with a particular focus on Sustainable Development Goal (SDG) 2.

## Methods

### Data source

This analysis utilized the Bangladesh MICS dataset from 2019, which is a national representative survey and collects information on key indicators related to the SDGs. The Bangladesh Bureau of Statistics (BBS) supervised this survey with financial support from UNICEF in Bangladesh.^17^

### Sample design and sample size

**Figure 1.**
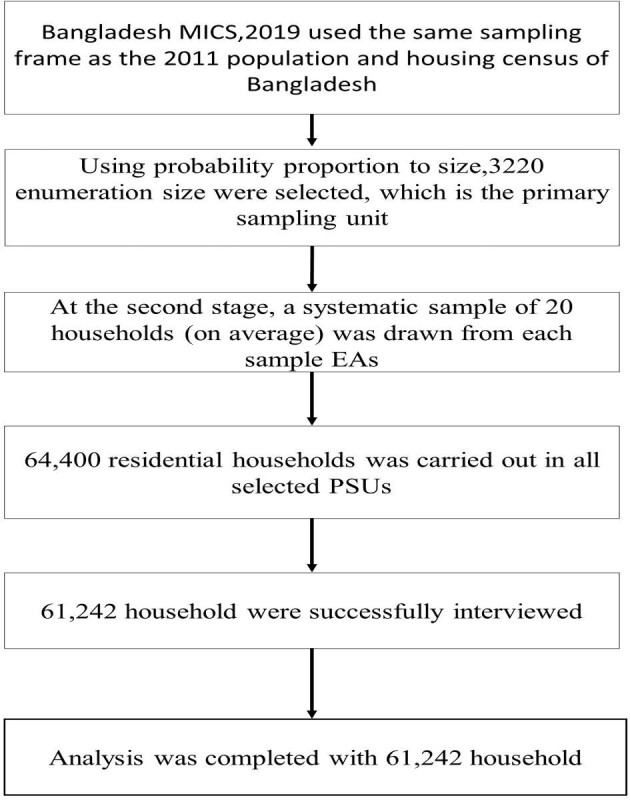
Procedure for final sample selection in this analysis.

The Bangladesh MICS 2019 used two-stage stratified cluster sampling and the 2011 Census of Population and Housing sample frame. Between these two stages, 3220 enumeration areas (EAs) were defined at the first stage and 20 households were drawn from each EA in the second stage. A large number of households (64 400 households) were initially selected for interviews and 61 602 households were successfully interviewed. To ensure an accurate representation of the survey results at the national level, all analyses were performed using appropriate sampling weights conforming to MICS guidelines.^17^ The Bangladesh MICS 2019 sample, despite its representativeness, uses varying sampling fractions within districts, requiring the computation of sample weights for accurate and unbiased results in the analysis of the survey data. The sample size for this analysis consisted of 61 242 households from across Bangladesh, with 13 564 households in urban areas and 47 678 households in rural areas.^17^ Figure 1 illustrates the overall procedure for sample analysis.

### Dependent variable

The target variable of this analysis is adequacy of iodized salt. In the Bangladesh MICS 2019, rapid test kits for potassium iodate (KIO_3_) were used to test for the presence of iodine salt used for cooking in the household.^17^ The MICS involves interviewers using salt iodization test kits to assess iodine levels in household salts. These kits contain a solution that causes a colour change in salt samples if they contain iodine. Each kit is designed to test at least 100 samples, with a noticeable blue or purple colour change indicating the presence of iodine when added to a small portion of iodized salt.^17^ According to the WHO, if the household used 15–40 ppm of iodine in salt, then it was considered as iodine adequate, otherwise not.^3^ The evaluation of the salt iodization test in the MICS 2019 was based on measurement values: 0 ppm (no reaction), 0–15 ppm and >15 ppm. Consequently, we transformed the salt iodization test results into a dichotomous variable to assess iodine adequacy. Specifically, households with at least 15 ppm iodine in the salt were labelled as ‘Yes’ for iodine adequacy, while those with <15 ppm iodine were labelled as ‘No’ for iodine adequacy. Thus the salt iodization test outcome was recoded as a dichotomous variable in order to measure the adequacy status of iodine in salt as follows: 1 if household level contains 15–40 ppm of iodine in salt and 0 if household level contains<15 ppm of iodine in salt.

### Independent variables

This study examined independent variables identified through a literature search^11,18–22,34–39^ and variable availability of the MICS dataset. For clarity, we categorized the explanatory variables into two main groups: individual-level factors and community-level factors. Women's age (15–19, 20–34, 35–49 y), women's education (no education, primary, secondary and above), household head's education (no education, primary, secondary and above), religion (Muslim, non-Muslim), child death (0, 1, ≥2), children ever born (CEB; 0, 1–2, ≥3), overall happiness (happy, neutral, unhappy), maternal functional difficulty (yes, no), sex of household head (male, female), ethnicity (Bengali, other), household size (<4, 4–5, ≥6) and wealth quintile (poor, middle, rich) were selected as individual-level factors for this analysis. The wealth index is a measure of wealth created using principal component analysis,^23^ ranking households based on their assets and final factor ratings. It is crucial for household health, targeting poverty alleviation programs and cross-tabulating with other variables. In Bangladesh, the Bangladesh Wealth Index incorporates 25 variables from the Bangladesh MICS 2019, with the poor category formed by merging the poorest and poorer groups and the rich category formed by combining the richest and richer groups.^17^ Further details regarding the construction of the wealth index can be found in other research.^24,25^

We consider residence (urban and rural), division (Barishal, Chattogram, Dhaka, Khulna, Mymensingh, Rajshahi, Rangpur, Sylhet) and community mass media exposure (low, high) as community-level factors. Community-level variables were derived by aggregating individual factors of interest within each cluster. The aggregates were calculated based on the proportion of a specific subcategory variable and in some cases the distribution of these generated variables was not normal. However, all community-level variables were divided into two groups using the median.^26^ Additionally, community-level media exposure was assessed by determining the proportion of households that had been exposed to at least one form of media, such as television, radio or newspaper. This was coded as 0 for communities with low media exposure (where <50% of households in the cluster had been exposed to at least one media source) and 1 for communities with high community-level media exposure (where ≥50% of households in the cluster had been exposed to at least one media source).^27^

### Statistical analysis

This study used various statistical methods to achieve its objectives. The analytical section was divided into three parts as univariate, bivariate and multivariate. In the univariate part, frequency distribution was used to summarize the independent variables along with percentage.

### Inequality analysis

To investigate socio-economic disparities, we utilized a concentration index as a measuring tool. It was defined as


\begin{eqnarray*}CIX = \frac{{2{\mathrm{cov}}\left( {{D}_i{\mathrm{|}}{R}_i} \right)}}{{\bar{D}}},
\end{eqnarray*}

where *CIX* is the concentration index, ${R}_i$ denotes the fractional rank within the socio-economic position distribution, ${D}_i$ is the index for the dependent variable and $\bar{D}$ is the mean of the outcome variable in the sample.

### Multilevel logistic regression analysis

In a multivariate setting, multilevel logistic regression is used to analyse and examine the influence of individual and community-level factors on the adequacy of iodized salt. In research studies, multilevel logistic regression models are frequently employed to account for the hierarchical nature of data and examine how individual- and group-level factors influence an outcome variable.^23^ In this study, the individual was nested within the household and the household was nested within the community. Given that the MICS dataset exhibits a community-level impact, it necessitates the adoption of a multilevel modelling approach rather than a single-level analysis.^28,29^ So, based on these nested criteria, a two-level logistic regression model is suitable for this study^30^ and the census enumeration cluster was considered as a random effect.

We fitted three models to examine influencing factors affecting iodine adequacy:

1. Empty (model 1): random intercept only
2. Individual level (model 2): all individual factors
3. Community level (model 3): both individual- and community-level factors

Fixed effects were presented as adjusted odds ratios (ORs) and 95% confidence intervals (CIs). Akaike's information criterion (AIC), Bayesian information criterion (BIC) and deviance were used to measure model fit. Lower values of AIC, BIC and deviance indicate a better fit than the former model.

The general hierarchical form of the two-level model can be written as^31^


\begin{eqnarray*}
Y = ( {\alpha + \beta {X}_{\scriptstyle{individual}}} ) + ( {\gamma {X}_{\scriptstyle{community}} + u + \epsilon }),
\end{eqnarray*}

where *Y* is the dependent variable, $\alpha $ is the intercept, $\beta ,\gamma $ are the effect of individual and community covariates, respectively, and $u,\epsilon $ are the level 2 and level 1 random effects, respectively.

Proportional change in variance (PCV) can be calculated to assess the contribution of each set of predictors (model 2 and 3) in explaining the variance in the outcome variable compared with the null model.^23^ The intraclass correlation coefficient (ICC) should be calculated before using multilevel models; if the ICC is >0, use multilevel logistic regression.^23,32^ The mathematical formula for the ICC was


\begin{eqnarray*}ICC = \frac{{{\sigma }^2}}{{{\sigma }^2 + \frac{\pi }{3}}}.
\end{eqnarray*}

Data management and analysis for this study were conducted using SPSS version 25 (IBM, Armonk, NY, USA) and R version 4.0.0 (R Foundation for Statistical Computing, Vienna, Austria). The interactive map with spatial analysis was created using the Leaflet package in R (version 4.2.2).

## Results

### Background characteristics of the household

Table 1 represents the background characteristics of the participants, with the highest portion (25.3%) of respondents from Dhaka and only 6% from Sylhet. More than two-thirds (77.9%) of respondents were from rural areas of Bangladesh. Most of the respondents (87.3%) were from male-headed households, almost all (98.8%) were Bengali and most (42.2%) of the women were between 20 and 34 y of age. About one-third of household heads (35%) had no education and one-third of household heads had a secondary or above education. Nearly half of the women (46%) had a secondary or above education. Most of the respondents (41.4%) belong to the poor wealth quintile. Nearly half of the respondents (47.0%) had a household size between four and five. More than half of the respondents (56.8%) had high community-level mass media exposure. Most of the respondents (78.3%) had no maternal functional difficulties. More than two-thirds of participants (73.5%) had no child death. Two-thirds of respondent (69.8%) were overall happy.

**Table 1.** Background characteristics of the study participants

Variables	Frequency	Percentage
Individual level factors		
Sex of household head		
Male	53 460	87.3
Female	7782	12.7
Ethnicity		
Bengali	60 527	98.8
Others	715	1.2
Religion		
Muslim	55 261	90.2
Non-Muslim	5981	9.8
Women age group (years)^a^		
15–19	4054	6.6
20–34	25 819	42.2
35–49	21 268	34.7
Household head education		
No education	21 459	35.0
Primary	16 587	27.1
Secondary and above	23 196	37.9
Women education^a^		
No education	9674	15.8
Primary	13 281	21.7
Secondary and above	28 185	46.0
Wealth quintile		
Poor	25 373	41.4
Middle	11 895	19.4
Rich	23 974	39.1
Household size		
<4	20 894	34.1
4–5	28 758	47.0
≥6	11 590	18.9
Maternal functional difficulty		
Yes	1599	2.6
No	47 974	78.3
Child death		
0	45 004	73.5
1	4998	8.2
≥2	1138	1.9
Child ever born^a^		
0	5989	9.8
1–2	25 786	42.1
≥3	19 365	31.6
Overall happiness^a^		
Happy	42 753	69.8
Neutral	6065	9.9
Unhappy	2315	3.8
Community-level factors		
Division		
Barishal	1388	5.7
Chattogram	10 736	17.5
Dhaka	15 512	25.3
Khulna	7290	11.9
Mymensingh	4561	7.4
Rajshahi	8745	14.3
Rangpur	7229	11.8
Sylhet	3681	6.0
Residence		
Urban	13 564	22.1
Rural	47 678	77.9
Community-level mass media exposure		
Low	26 444	43.2
High	34 798	56.8

a Some missing observations.

### District-wise household iodine adequacy status in Bangladesh

**Figure 2.**
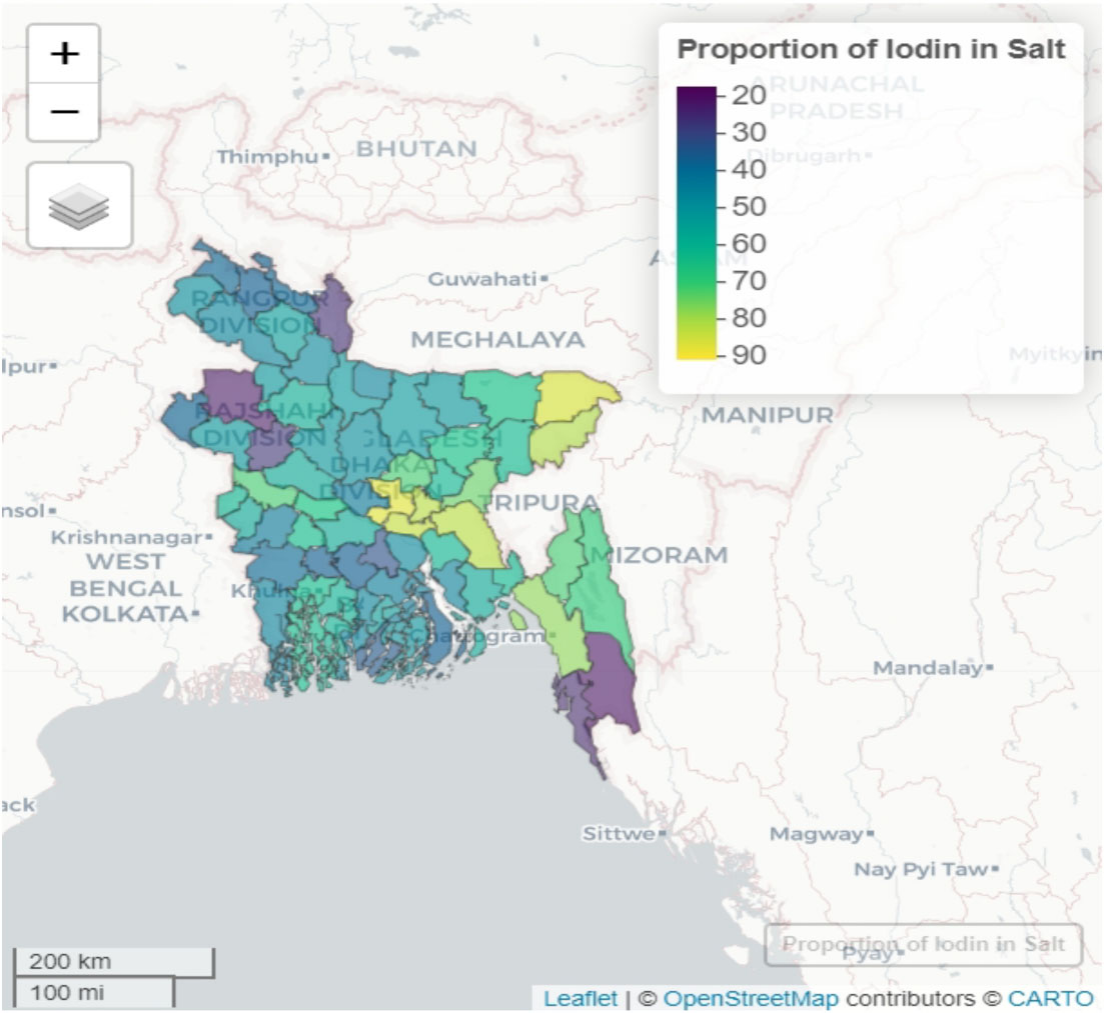
Geographical map of district-wise household iodine concentration status in Bangladesh.

Among the districts in Bangladesh, Dhaka had the highest proportion of iodine adequacy status, with 90.7% of households meeting the adequacy criteria (Figure 2). Following Dhaka, Sylhet had the second-highest proportion of iodine adequacy, with 89.6% of households meeting the criteria. Munshiganj (86.7%), Narayangonj (85.6%), Cumila (85.1%) and Maulvibazar (83.4%) districts also exhibited relatively high iodine adequacy. In contrast, the districts with the lowest iodine adequacy status were Bandarban (17.6%), Naogaon (19.1%) and Kurigram (23.4%). These districts have a significantly lower proportion of households meeting the iodine adequacy criteria.

### Prevalence of adequacy of iodine salt at the household level with individual- and community-level factors

The study revealed that the overall prevalence of iodine adequacy was 57.8% (95% CI 57.4 to 58.2). As seen in Table 2, the prevalence of iodine adequacy in salt was notably higher in Sylhet, at 76.5%. The analysis also indicated a significantly higher prevalence of iodine adequacy in household salt in urban areas (77.9%) compared with rural areas (52.2%). Additionally, the prevalence of iodine adequacy in salt varied among different subgroups: for female-headed households, it was 62.3%, for non-Muslim households, it was 59.8%, and for Bengali ethnic groups, it was 57.7%. Notably, the highest prevalence of iodine adequacy was observed among women 20–34 y of age (60.9%), households led by individuals with a secondary education or higher (70.2%) and women with a secondary education or higher (66.1%). The highest prevalence of iodine adequacy was observed among respondents from the rich wealth quintile, at 79.2%. Those with a household size between four and five had a prevalence of 58.1%. At the community level, households with high mass media exposure exhibited a prevalence of 66.2%, while those without maternal functional difficulties showed a prevalence of 59.8%. Regarding individual characteristics, respondents who had not experienced child death had a prevalence of 60.3%, those who had not given birth to children had a prevalence of 62.4% and those who reported overall happiness in their lives had a prevalence of 61.4% when it came to using iodized salt in their daily lives.

**Table 2.** Prevalence of adequacy of iodine salt at the household level by individual- and community-level factors

Variables	Prevalence (%)	95% CI
Community-level factors		
Division		
Barishal	37.9	36.3 to 39.5
Chattogram	67.5	66.6 to 68.3
Dhaka	69.2	68.5 to 69.9
Khulna	53.4	52.3 to 54.6
Mymensingh	50.8	49.4 to 52.2
Rajshahi	44.1	43.1 to 45.2
Rangpur	41.0	39.9 to 42.2
Sylhet	76.5	75.2 to 77.8
Residence		
Urban	77.9	77.2 to 78.6
Rural	52.2	51.8 to 52.6
Community-level mass media exposure		
Low	46.6	46.0 to 47.2
High	66.2	65.7 to 66.7
Individual-level factors		
Sex of household head		
Male	57.1	56.7 to 57.5
Female	62.3	61.2 to 63.4
Ethnicity		
Bengali	57.7	57.4 to 58.1
Others	61.9	57.6 to 66.0
Religion		
Muslim	57.6	57.1 to 58.0
Non-Muslim	59.8	58.6 to 61.1
Women age group (years)		
15–19	56.9	55.4 to 58.4
20–34	60.9	60.3 to 61.5
35–49	58.0	57.4 to 58.7
Household head education		
No education	45.9	45.3 to 46.6
Primary	56.0	55.3 to 56.8
Secondary and above	70.2	69.6 to 70.7
Women education		
No education	47.0	46.0 to 48.0
Primary	54.3	53.5 to 55.1
Secondary and above	66.1	65.5 to 66.6
Wealth quintile		
Poor	38.7	38.1 to 39.3
Middle	55.1	54.2 to 55.9
Rich	79.2	78.7 to 79.8
Household size		
<4	57.6	56.9 to 58.2
4–5	58.1	57.6 to 58.7
≥6	57.3	56.4 to 58.2
Maternal functional difficulty		
Yes	51.2	48.8 to 53.6
No	59.8	59.4 to 60.3
Child death		
0	60.3	59.8 to 60.7
1	52.8	51.5 to 54.2
≥2	53.1	50.2 to 55.9
Child ever born		
0	62.4	61.2 to 63.6
1–2	61.9	61.3 to 62.4
≥3	55.3	54.6 to 55.9
Overall happiness		
Happy	61.4	61.0 to 61.9
Neutral	50.1	48.8 to 51.3
Unhappy	47.1	45.1 to 49.1
Total	57.8	57.4 to 58.2

### Socio-economic inequalities of the adequacy of iodine salt at the household level

**Figure 3.**
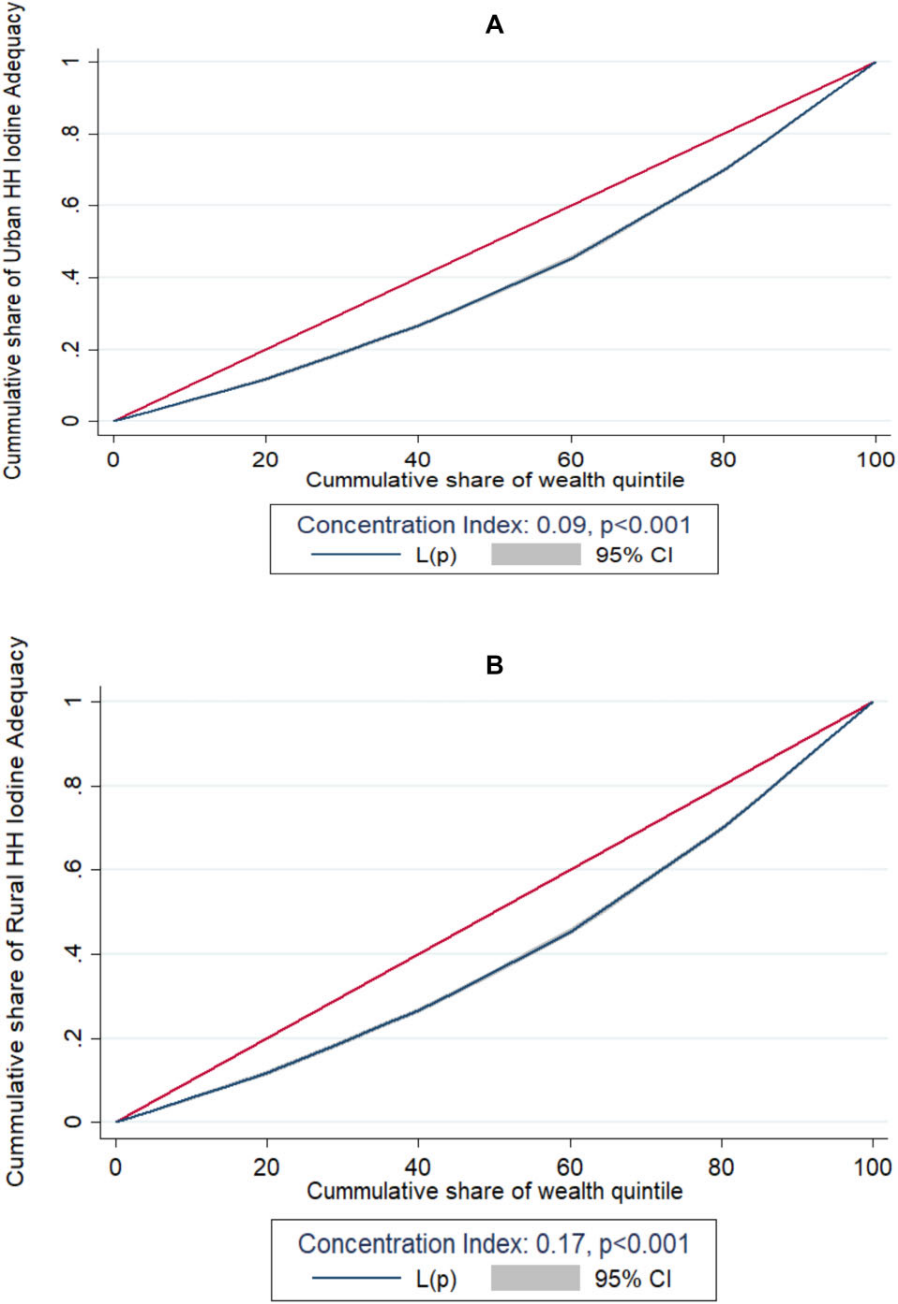
Socio-economic inequalities of **(A)** urban and **(B)** rural groups for household iodine adequacy status in Bangladesh.

In the case of urban areas, the concentration index value of 0.09 (p<0.001) suggests that iodine adequacy in household salt is concentrated among the two highest wealth quintile groups. This indicates that wealthier households in urban areas are more likely to have iodine-adequate salt compared with lower-income households (Figure 3). In contrast, in rural areas the concentration index value of 0.17, which is slightly higher than the value for urban areas, indicates that iodine adequacy in household salt is even more concentrated among the two highest wealth quintile groups. In other words, the wealthier and relatively wealthier households in rural areas have a greater likelihood of having access to iodine-adequate salt, while the poorer and poorest households are less likely to have access to iodine-adequate salt.

### Individual- and community-level factors related to the adequacy of iodine salt in households

#### Random effects and model comparison

The null model establishes an intercept-only model to assess the appropriateness of estimating random effects at the cluster level (Table 3). The null model exhibited an ICC of 33%, indicating substantial heterogeneity between individual- and community-level factors. The ICC values for models 2 and 3, respectively, are 25% and 20%. Furthermore, in model 3, the highest PCV value was 51.22%. This suggests that factors at both the individual and community levels can account for 51.22% of the variation in the iodine adequacy of salt among households. To assess model comparisons and fitness, we utilized the AIC and BIC. Among the tested models, model 3 displayed the lowest AIC, BIC and deviance values, indicating that it was the most suitable and best-fitting model.

**Table 3.** Random effects and model comparison for factors associated with adequacy of iodine in salt among households in Bangladesh

Parameter	Model 1	Model 2	Model 3
ICC (%)	33.0	25.0	20.0
PCV (%)	–	31.71	51.22
Model fitness			
AIC	66 848.1	63 170.1	62 341.6
BIC	66 866.2	63 342.4	62 595.7
Deviance	62 553.2	61 016.4	60 122.8

#### Fixed effects results

Table 4 displays the outcomes of a two-level logistic regression analysis, demonstrating the fixed effects of iodine adequacy status for individual and community characteristics.

**Table 4.** Adjusted ORs with fixed effects of selected covariates for the iodine concentration in salt among households in Bangladesh, obtained from two-level random intercept multilevel logistic models

	Model 2		Model 3	
Variables	aOR	95% CI	aOR	95% CI
Fixed effects				
Individual-level factors				
Women's age (years)				
15–19	0.73***	0.65 to 0.81	0.70***	0.62 to 0.78
20–34	0.94***	0.89 to 1.00	0.90***	0.85 to 0.95
35–49 (ref.)	1		1	
Womens’ education				
No education	0.83***	0.78 to 0.89	0.80***	0.74 to 0.85
Primary	0.93*	0.88 to 0.99	0.91**	0.86 to 0.96
Secondary or higher (ref.)	1		1	
Wealth quintile				
Poor	0.27***	0.26 to 0.29	0.33***	0.31 to 0.35
Middle	0.46***	0.43 to 0.49	0.52***	0.49 to 0.55
Rich (ref.)	1		1	
Religion				
Muslim	0.85**	0.78 to 0.93	0.89*	0.82 to 0.98
Non-Muslim (ref.)	1		1	
Overall happiness				
Happy	1.39***	1.27 to 1.52	1.34***	1.22 to 1.47
Neutral	1.16**	1.04 to 1.29	1.12*	1.01 to 1.25
Unhappy (ref.)	1		1	
Maternal functional difficulty				
Yes	0.80**	0.70 to 0.91	0.81**	0.71 to 0.92
No (ref.)	1		1	
Sex of household head				
Male	0.95	0.89 to 1.01	0.98	0.91 to 1.04
Female (ref.)	1		1	
Household head education				
No education	0.63***	0.59 to 0.66	0.62***	0.59 to 0.66
Primary	0.78***	0.73 to 0.82	0.77***	0.73 to 0.82
Secondary or higher (ref.)	1		1	
Child death				
0	0.89*	0.81 to 0.99	0.83***	0.75 to 0.92
1	0.85**	0.75 to 0.95	0.79***	0.71 to 0.89
≥2 (ref.)	1		1	
Child ever born				
0	1.23***	1.11 to 1.35	1.21***	1.10 to 1.33
1–2	1.16***	1.09 to 1.23	1.19***	1.12 to 1.26
≥3 (ref.)	1		1	
Community-level factors				
Residence				
Urban			2.08***	1.88 to 2.31
Rural (ref.)			1	
Division				
Barishal			0.17***	0.14 to 0.21
Chattogram			0.42***	0.35 to 0.51
Dhaka			0.44***	0.37 to 0.52
Khulna			0.25***	0.21 to 0.30
Mymensingh			0.32***	0.26 to 0.39
Rajshahi			0.17***	0.14 to 0.21
Rangpur			0.20***	0.16 to 0.24
Sylhet (ref.)			1	
Community-level mass media exposure				
Low			0.81***	0.77 to 0.85
High (ref.)			1	

ref.: reference category.

*p<0.05, **p<0.01, ***p<0.001.

A multivariable two-level mixed effects model evaluating the relationship between Bangladesh's iodine sufficiency status and different levels of components is shown in Table 4. In the individual-level model (model 2), only individual-level covariates were included, such as women's age group, women's education, wealth quintile, religion, overall happiness, maternal functional difficulty, sex of the household head, household head education, child death and number of children ever born. However, in this section we will focus on providing a detailed description of the final model, model 3. Model 3 incorporates community-level factors including residence (urban or rural), division and access to mass media.

In accordance with the final model from Table 4, younger women had a lower chance of adequate iodine concentration then older women in Bangladesh. More specifically, women ages 15–19 y had a 30% lower chance of having adequate iodine in household salt compared with women ages 35–49 y (adjusted odds ratio [aOR] 0.70 [95% CI 0.62 to 0.78]). Compared with secondary and above educated women, uneducated and primary educated women were 20% (aOR 0.80 [95% CI 0.74 to 0.85]) and 9% (aOR 0.91 [95% CI 0.86 to 0.96]) less likely to have adequacy of iodized salt, respectively.

The study identified a significant association between the wealth quintile and the iodine adequacy in household salt. The OR increased as respondents’ wealth quintile increased, indicating a positive relationship between wealth and the likelihood of having adequate iodine salt. Specifically, compared with the rich group of respondents, those classified as poor had 67% lower odds of having adequate iodine salt in their households (aOR 0.33 [95% CI 0.31 to 0.35]). Similarly, respondents from the middle wealth quintile had 48% lower odds of having adequate iodine salt compared with the rich group (aOR 0.52 [95% CI 0.49 to 0.55]).

Regarding the overall happiness of respondents, those who reported feeling happy had a 34% higher chance of using iodized salt compared with those who were unhappy (aOR 1.34 [95% CI 1.22 to 1.47]). In contrast, women who reported having any type of functional difficulty had a 19% lower likelihood of consuming iodized salt compared with the reference category (aOR 0.81 [95% CI 0.71 to 0.92]).

In Bangladesh, if the head of the household is uneducated, the likelihood of using iodized salt is 38% lower compared with households with an educated household head (aOR 0.62 [95% CI 0.59 to 0.66]). Women who had no child deaths had 17% lower odds of using iodized salt compared with women who had experienced two or more child deaths (aOR 0.83 [95% CI 0.75 to 0.92]). Similarly, women who had one child death had 21% lower odds of using iodized salt compared with those with two or more child deaths (aOR 0.79 [95% CI 0.71 to 0.89]). Women who had never given birth to a child were 1.21 times more likely to have iodine adequacy in salt compared with women with three or more children. Similarly, women who had one to two children were 1.19 times more likely to have adequacy of iodized salt compared with women who had three or more children.

At the community level, residence was found to have a significant relationship with the concentration of iodine in salt. Participants residing in urban areas were 2.08 times more likely to use iodized salt compared with participants from rural areas. Households from Barishal had 83% lower odds of using iodized salt compared with households from Sylhet (aOR 0.17 [95% CI 0.14 to 0.21]). Similarly, households from Dhaka division had 56% lower odds of using iodized salt compared with households from Sylhet (aOR 0.44 [95% CI 0.37 to 0.52]). Households with low community-level mass media exposure were 19% less likely to consume iodized salt compared with those who had high community-level mass media exposure (aOR 0.81 [95% CI 0.77 to 0.85]).

## Discussion

The primary objective of this study was to examine the association between the adequacy of iodized salt in households and various socio-economic factors in Bangladesh. Utilizing the 2019 Bangladesh MICS data, we employed a multilevel logistic regression model for our analysis. The results revealed several significant factors, including religion, physical disability and exposure to media, that equally influence the presence of iodized salt intake. Additionally, women's age, wealth status, education level and the education of the household head were identified as positive contributors to the adequacy of iodine in household salt. These findings emphasize the need for careful consideration of these variables in the formulation of policies related to iodized salt consumption.

A negative association was observed between women's age and adequate iodine salt intake, revealing that younger women in Bangladesh had a lower likelihood of achieving sufficient iodine concentrations compared with their older counterparts. Although specific references are not readily available in the existing literature, a plausible explanation lies in age-related variations in dietary practices, reproductive stages and nutritional awareness. Previous research indicates that periods of rapid growth, such as adolescence and pregnancy, heighten iodine requirements.^33^ Younger women, especially those of reproductive age, may face increased iodine needs due to pregnancy and lactation, crucial for foetal brain development and breastfeeding.^33^ These life stages can impose greater demands for iodine, and insufficient dietary practices or awareness among younger women may contribute to lower iodine concentrations. Conversely, older women, particularly postmenopausal individuals, may exhibit stable or reduced iodine needs. Hormonal changes during menopause and potentially more health-conscious dietary habits in older age could result in a higher likelihood of adequate iodine concentrations. However, it should be noted that a prior study conducted in Bangladesh^11^ yielded contrasting results, indicating a significantly higher likelihood of iodized salt access among young women, emphasizing the need for further research on this issue.

The findings of this study indicate that the education levels of the household head and of the women residing in the household are the most significant factor in consuming sufficient iodized salt. This aligns with previous studies conducted in Bangladesh.^11^ Educated individuals tend to be more aware of the negative effects of iodine deficiency and are therefore more willing to use iodized salt for their families. According to the literature, there is evidence to suggest that developing nations are experiencing more concerning signs due to the increase in iodine deficiency and urinary iodine concentration during pregnancy, which is influenced by women's level of education.^34,35^ In addition, it appears that women in Bangladesh bear a greater responsibility for making household decisions and are typically responsible for preparing meals for their families.^36^ As a result, they tend to place a greater emphasis on nutrition, which underscores the importance of educating women in this area.

These findings highlight the disparities in iodine adequacy in household salt across different socio-economic groups, with wealthier households generally having better access to iodine-adequate salt compared with lower-income households, particularly in rural areas. The place of residence and wealth status were identified as significant factors in consuming adequate iodized salt in individual households in Bangladesh. The availability and accessibility of iodized salt can vary depending on the location.^37^ In urban areas and well-connected regions, iodized salt may be more readily available and accessible to households. However, in remote rural areas or areas with limited infrastructure, access to iodized salt can be challenging. The lack of nearby markets or limited transportation options can make it difficult for households in these areas to obtain iodized salt.^37,38^

The socio-economic status of households can differ based on their place of residence. In low-income or impoverished areas, households may have limited financial resources to purchase iodized salt, which can be relatively more expensive than non-iodized salt.^39^ Affordability can affect the ability of households to choose and purchase iodized salt regularly. Moreover, awareness about the importance of iodized salt and its role in preventing IDDs may vary across different regions. In some areas, there may be a lack of knowledge about the benefits of iodized salt or insufficient awareness campaigns and educational programs regarding its use. This can lead to lower demand and use of iodized salt in those areas. The iodine status showed significant variations across socio-economic strata, with higher urinary iodine levels observed in the low socio-economic group, while the high socio-economic group exhibited the lowest urinary iodine levels.^13^

Religious beliefs and practices can influence dietary habits, including the consumption of iodized salt.^40^ In Bangladesh, where Islam is the predominant religion, cultural and religious practices may impact the preference for certain food items, including salt. Understanding the religious context and incorporating religious leaders and institutions in promoting the importance of iodized salt within religious guidelines can contribute to increased consumption.^40^

The number of children ever born in a household is also identified as an influencing factor for the consumption of iodized salt.^41^ Households with a greater number of children are more likely to prioritize their nutritional needs, including the use of iodized salt, as iodine deficiency can have adverse effects on child development.^41^ Additionally, households with more children may have a greater awareness of the importance of iodized salt due to previous experience or exposure to healthcare providers.

The level of overall happiness or subjective well-being within households plays a pivotal role in shaping health-related behaviours, extending its influence on the consumption patterns of iodized salt. Extensive research has consistently demonstrated a positive correlation between heightened happiness levels and the adoption of healthier behaviours, particularly in terms of maintaining a well-balanced diet.^42^ Individuals experiencing greater overall happiness are inclined to make health-conscious choices, which may include a heightened awareness and motivation to actively incorporate iodized salt into their dietary practices. This connection between subjective well-being and iodized salt consumption emphasizes the broader impact of emotional well-being on health behaviours, emphasizing the potential role of positive emotions in fostering nutritional awareness and encouraging habits that contribute to improved health outcomes. As such, considering the interplay between happiness and dietary choices provides valuable insights into the complex dynamics influencing iodized salt intake at the household level.

Among all the participants, those living in urban areas were more likely to use iodized salt compared with participants residing in rural areas. Rural areas sell twice as much open salt as urban areas, attracting poorer residents to opt for cheaper options.^37^ Rural residents in Bangladesh are comparatively poorer than urban ones, which is exacerbated by a lack of general awareness and education, leading to the consumption of low-quality salt.^43^

Exposure to community-level mass media, such as television, radio and print media, plays a crucial role in disseminating information and raising awareness about iodized salt.^44^ Individuals with higher levels of mass media exposure are more likely to be aware of the benefits of iodized salt and its role in preventing IDDs.^44^ Access to mass media, particularly in urban areas, provides opportunities for communication campaigns and education programs to promote iodized salt consumption.

Our identified factors collectively contribute to regional disparities in the consumption of iodized salt in individual households in Bangladesh. Efforts to address these challenges include improving availability and accessibility, raising awareness, promoting affordability and strengthening policy implementation to ensure consistent access to iodized salt across the country. The government plays a crucial role in promoting iodized salt consumption through policy interventions and programs. While national-level initiatives aim to ensure universal salt iodization, the implementation and monitoring of these programs can vary across different regions. In some cases, enforcement of regulations and quality control mechanisms may be weaker in some areas, leading to inconsistent adequacy and consumption of iodized salt.

This study on the adequacy of iodized salt in Bangladeshi households has both strengths and limitations. It lacks an assessment of critical variables such as the practice of using iodized salt,^45^ community education,^46^ community poverty^46^ and consumer satisfaction level.^47^ Additionally, due to secondary data, a cause-and-effect relationship cannot be established. However, the study offers a contemporary perspective on factors influencing iodized salt adequacy at both the individual and community levels. The study uses robust analytical methods, including a two-level logistic model, and utilizes nationally representative data with a substantial sample size of 61 242 households. This national survey–based approach can provide valuable insights for policymakers and stakeholders in formulating intervention strategies at both the individual and community level.

## Conclusions

Our study presents a comprehensive analysis of factors influencing iodized salt adequacy in Bangladeshi households. We identified several key contributors that are required for policy implication. Notably, younger women exhibited lower iodine concentrations, hinting at nuanced interactions with age-related dietary and reproductive factors. The positive impact of household head and women's education on iodized salt adequacy underscores the pivotal role of education in promoting health-conscious behaviours. Disparities in wealthier households, particularly in rural settings, and regional variations shed light on challenges in ensuring equitable access, influenced by both infrastructure and cultural factors. Additionally, the study highlights the importance of considering factors such as the number of children, overall happiness and media exposure in shaping iodized salt consumption patterns. Addressing these challenges necessitates targeted interventions and effective policies, with a crucial role for government involvement. Initiatives focusing on education improvement, awareness campaigns and economic measures can effectively combat iodine deficiency. This comprehensive strategy will contribute to mitigating iodine deficiency and associated health risks.

## Data Availability

Data from the 2019 Bangladesh MICS are available from http://mics.unicef.org/.
